# Nurses’ Experiences of Caring for Patients with COVID-19: A Qualitative Study

**DOI:** 10.1177/21582440221144982

**Published:** 2022-12-24

**Authors:** Maysa H. Almomani, Wejdan A. Khater, Laila M. Akhu-Zaheya, Aladeen Alloubani, Safa A. AlAshram, Mohammed Azab, Adeeb K. Al-malkawi

**Affiliations:** 1 Department of Adult Health Nursing, Faculty of Nursing, Jordan University of Science and Technology, Irbid, Jordan; 2 Nursing Research Unit, King Hussein Cancer Center, Amman, Jordan; 3 Specialization Records Department, Jordanian Nursing Council, Amman, Jordan; 4 Department of Basic Medical Sciences, Faculty of Medicine, The Hashemite University, Zarqa, Jordan; 5 Prince Hamza Hospital, Amman, Jordan

**Keywords:** COVID-19, nurses, experiences, ability, willingness

## Abstract

The purpose of this study was to explore nurses’ experiences, abilities, and willingness to care for patients with Coronavirus Disease 2019 (COVID-19). A descriptive qualitative study was conducted among 12 nurses working with patients with COVID-19. Purposive sampling was used to recruit participants from two national hospitals in Jordan. Semi-structured interviews (45–90 minutes each) with open-ended questions were held via Zoom to collect data. Four major themes emerged from the data analysis. The first theme, uncertainty, consisted of two subthemes: new experience and lack of training. The second theme was related to social stigma by society and other staff members. The third theme of front-line fighters consisted of two subthemes: empowering the main health caregiver and community acknowledgment. The fourth theme was related to challenges and consisted of two subthemes: physical and psychological challenges. At the beginning of the outbreak of COVID-19, the nurses had experienced a lack of certainty, physical and psychological challenges, and social stigmatization, which had negatively affected their willingness and ability to fight the outbreak. However, the nurses reported growing professionally and psychologically with time and becoming more knowledgeable, skillful, powerful, and confident care providers during the pandemic. Being able to fulfill their responsibilities and being acknowledged by others gave the nurses a sense of achievement. Early education and training about COVID-19, clear infection control protocols and guidelines, psychological counseling, and adequate social support are essential steps for enhancing nurses’ mental well-being and willingness and ability to fight COVID-19.

## Introduction

In March 2020, the World Health Organization (WHO, 2020) declared the outbreak of the Coronavirus Disease 2019 (COVID-19) a global pandemic. As of January 4, 2022, a total of 290,959,019 confirmed cases and 5,446,753 deaths had been reported globally. In Jordan, the number of COVID-19 cases is spreading rapidly, reaching 1,067,253 confirmed cases and 12,742 deaths, as of January 4, 2022 (WHO, 2022). Therefore, the disease is considered highly infectious with a substantial fatality rate (Shi et al., 2020), which has significant health and psychosocial impacts on various constituents of society (Billings et al., 2021; Garbóczy et al., 2021; Khatatbeh, Khasawneh, et al., 2021). The burden of COVID-19 overwhelmed the healthcare systems and healthcare workforce (Khatatbeh, Alhalaiqa, et al., 2021). The acute care hospitals and the entire healthcare system are overwhelmed with infected patients, thus escalating the demand for the nursing workforce and workloads (Iheduru-Anderson, 2021), and causing various physical, social-psychological, and emotional challenges for nurses in coping with work demands (Billings et al., 2021; Chau et al., 2021; Galehdar et al., 2021; Halcomb et al., 2020; Huang et al., 2020; Khatatbeh, Alhalaiqa, et al., 2021; Liu et al., 2020).

Healthcare providers (HCPs), including nurses, play a vital role in response to pandemics and are at the front-line of exposure to infection (Huang et al., 2020; Liu et al., 2020; Shi et al., 2020). Nurses are directly involved in caring for patients with COVID-19, which makes them more susceptible to becoming infected and transmitting this highly contagious and lethal virus among their families and colleagues (Galehdar et al., 2021; Halcomb et al., 2020; Huang et al., 2020; Khatatbeh, Alhalaiqa, et al., 2021; Liu et al., 2020). Therefore, nurses must adhere to strict infection prevention and control guidelines, which may expose them to significant physical and psychological burdens (Billings et al., 2021; Galehdar et al., 2021; Huang et al., 2020; Kackin et al., 2021; Khatatbeh, Alhalaiqa, et al., 2021; Villar et al., 2021). Nurses who have worked with COVID-19 have reported experiencing fear and stress of contracting and transmitting the virus, uncertainty by working with new frequently changing protocols, and undergoing physical exhaustion caused by overwhelming workloads and use of personal protection equipment (PPE) (Billings et al., 2021; Dubey et al., 2020; Galehdar et al., 2021; Huang et al., 2020; Kackin et al., 2021; Liu et al., 2020). These negative experiences with the unique environment and workflow may be compounded by depletion of PPE and resources and inadequate information about the virus and treatments (Billings et al., 2021; Galehdar et al., 2021; Iheduru-Anderson, 2021; Khatatbeh, Alhalaiqa, et al., 2021).

In addition, nurses’ experiences of caring for COVID-19 patients may vary; from feelings of sadness, anxiety, powerlessness, and guilt by witnessing patients’ suffering, fears, and death (Galehdar et al., 2021; Huang et al., 2020; Liu et al., 2020; Rathnayake et al., 2021) to the feeling of satisfaction at the end of their duty (Rathnayake et al., 2021). Further, nurses’ personal and social life is affected by prolonged working hours, separation from family, isolation, social stigmatization, negative media coverage, and lack of adequate support (Billings et al., 2021; Chau et al., 2021; Khatatbeh, Alhalaiqa, et al., 2021). Consequently, nurses may experience psychological and emotional problems such as fear, stress, anxiety, depression, and insomnia (Brooks et al., 2018; Dubey et al., 2020; Huang et al., 2020; H. S. Kang et al., 2018; Liu et al., 2020; Xiong & Peng, 2020), impacting their ability to concentrate, make decisions, and maintain their physical and psychological well-being (Preti et al., 2020; Xiong & Peng, 2020).

The ability of healthcare systems to cope during a pandemic depends greatly on nurses and their willingness and ability to work (Ives et al., 2009). Nurses may be torn between caring for patients while also trying to maintain their health to protect their families and communities (Brucker, 2020), which may impact their performance and willingness to work (Shi et al., 2020) or lead them to consider resigning (Brooks et al., 2018; Halcomb et al., 2020; Temsah et al., 2020). However, nurses’ ability and willingness to work during pandemics can be enhanced through adequate education, advanced training, provision of sufficient protective resources (Ives et al., 2009; Martin, 2011; Que et al., 2020; Shi et al., 2020; Taylor et al., 2018), provision of adequate guidance (Ives et al., 2009), and provision of psychological support (Taylor et al., 2018).

The available research focused mainly on exploring psychological and physical stress (Billings et al., 2021; Huerta-gonz et al., 2021; L. Kang et al., 2020; Xu et al., 2021), limited research assessed nurses’ abilities, and willingness to care for patients with COVID-19. In Jordan, little research has been conducted to solely explore the psychological impact among nurses not specified to be caring for COVID-19 patients (Hawari et al., 2021; Qutishat et al., 2021; Shahrour & Dardas, 2020). Only one Jordanian study explored the lived experience of nurses caring for COVID-19 patients (Khatatbeh, Alhalaiqa, et al., 2021). Effectively dealing with the COVID-19 pandemic requires the assessment of nurses’ experiences, willingness, and abilities to care for infected patients. In response to COVID-19, various countries, including Jordan, applied various strategies of reshaping hospitals and reallocating the HCPs and facilities to distribute the medical burden and overcome resources and personnel shortages (Her, 2020). The different changes may expose nurses to various challenges and affect their willingness and abilities to work with COVID-19. In addition, nurses’ experiences in Jordan may differ from others across countries due to differences in healthcare systems, resources, and cultural backgrounds. Understanding nurses’ experiences, abilities, and willingness increases the efficiency of healthcare systems in fighting COVID-19 and other infectious disease outbreaks (Y. Kim, 2018) and optimizes nursing workforce retention, sustainability, and care quality (Halcomb et al., 2020). Therefore, the present study aimed to explore nurses’ experiences, abilities, and willingness to care for patients with COVID-19.

## Method

### Study Design

A qualitative descriptive approach was used to gain an in-depth understanding of nurses’ experiences of caring for patients with COVID-19. The qualitative descriptive approach is descriptive, and it is widely used in examining health and nursing-related phenomena (Polit & Beck, 2021). The design is a widely mentioned research approach that has been identified as appropriate for research questions focused on exploring the who, what, and where of events or experiences and gaining insights from informants regarding a poorly understood phenomenon (H. Kim et al., 2017). In the current study, the qualitative approach focuses on answering “who,” “what,” and “where” questions related to nurses’ experiences of caring for patients with COVID-19.

### Sample and Setting

Purposive sampling was used to recruit twelve nurses working with patients with COVID-19. The inclusion criteria were based on nurses who provided direct care to patients diagnosed with COVID-19. Participants were recruited from one governmental hospital in the capital of Jordan and one educational hospital in the north of Jordan, both of which were receiving patients with COVID-19.

A list of nurses who provide direct care to patients with COVID-19 was obtained from the nursing administrative office to recruit eligible participants. A snowball sampling approach was then employed to reach more participants by asking each participant about other participants covering the study inclusion criteria. This procedure was continued until the researcher reached the data saturation level. Data saturation refers to the repetition of discovered information and confirmation of previously collected data (Speziale & Carpenter, 2011). After the 12 nurses were interviewed, no further coding was feasible, so we stopped recruiting nurses. Ten of these nurses were also part of a sample in another published study on COVID-19 (Alloubani et al., 2021).

### Interviews

Semi-structured video-recorded interviews with open-ended questions were held with 12 nurses via Zoom, with each interview lasting between 45 and 90 minutes. During the interviews, the interviewers noticed and recorded nonverbal responses. Verbal consent was obtained from the participants for audio-recording the interviews. The interviews were guided by open-ended questions and additional questions depending on the participants’ responses. When required, the interviewers asked follow-up questions to allow the participants to clarify their responses. Examples of questions included in interview guides (Table 1) are: would you please share with us your experience in caring for patients with COVID-19? How are nurses protected during the crisis of care during pandemics? What are the challenges nurses are facing when caring for patients with coronavirus? How does caring for patients with coronavirus affect nurses’ physical and psychological health, and what kind of support do nurses receive while caring for patients with coronavirus?

**Table 1.** Interview Guide.

*At the beginning of the interview*: identifying socio-demographic data of the nurses, including age work experiences, marital status, experiences with COVID-19, and participants’ educational level.
- Would you please share with us your experience in caring for patients with COVID-19
- If you had previous experience with epidemics such as H1N1 influenza or SARS or any other infectious diseases, how is this situation different?
- Could you please explain to us the protocol or measures that have been taken to protect health care provided (nurses) during the caring of patients infected with COVID-19?
*Follow up*:
- From your perspective, what are other measures should be taken in these crises?
- From your perspective, what are the challenges nurses are facing when caring for patients with coronavirus?
*Follow up*:
- How are these challenges affecting the quality of care?
- Could you please describe the Isolation room?
- What do you think is the significant role of nurses in this situation, and how is this role different than other health care providers’ roles?
- What would you like to suggest for caring for a patient with COVID-19?
- How caring for patients with coronavirus would affect nurses’ physical health?
- How caring for patients with coronavirus would affect nurses’ psychological health?
- From your perspective, what are the most distressing/stressful or concerning issues in caring for a patient with COVID-19?
- What kind of support do nurses receive while caring for patients with coronavirus?
- If working with COVID-19 is voluntary, will you do it?
*Follow-up*:
- How do nurses decide whether to accept caring for a patient with COVID-19?
- What commitments do nurses have that might lead them to care for a patient with COVID-19?
*End of interview*:
- Is there anything you would like to add or thought I should ask you, but I did not?
- Do you have any questions for us?

*Note*. COVID-19 = coronavirus disease 2019; SARS = severe acute respiratory syndrome.

First, the interview was pilot tested with one nurse not included in the study sample. Then, the interviews were conducted by the first two authors during the nurses’ 14-day quarantine period. The interviews were held according to the nurses’ time preferences, with the interviewers in quiet rooms and the nurses alone in their own homes. None of the nurses refused to participate or withdrew from the study. The interviewers established rapport with the potential participants before their enrolment in the study and ensured to maintain this rapport with the participating nurses. The interviews were transcribed verbatim in Arabic, translated into English by the research team, checked by a qualified translator and one of the study nurses, and analyzed for thematic analysis.

### Ethical Considerations

This study was approved by the Institutional Review Board (IRB #131/132/2020) at a university in Jordan and the selected hospitals. The study purpose was explained, and verbal consent was obtained from the nurse before the interview. Nurses were assured their participation was voluntary and that they had the right to withdraw from the study at any time without consequences.

Pseudonyms were used to protect their confidentiality and privacy. Further, participants’ data were kept in locked files that the primary researcher could access only in a password-protected computer. Additionally, participants were informed that all tapes and transcribed interviews would be discarded after completing the study.

### Trustworthiness

The trustworthiness was assured through credibility, transferability, dependability, and confirmability (Lincoln & Guba, 1985). A “member checking” process was used to ensure the credibility of the data. Member check was done using two methods: first, at the end of each interview, the researcher summarized major points and comments, giving the participants a chance to validate and correct researcher misperception in their words. Second, after completing the study, two nurses who considered the emerging themes a reflection of their experiences were given a summary of the themes and the study’s abstract.

To ensure the transferability of the study findings, the researchers did their best to describe the study’s context accurately. Therefore, the reader can judge how applicable the results are to other situations or cultures. Further, transferability was assured through a thick and detailed description of the study themes and phenomenon.

All researchers’ dependability and confirmability during data collection and analysis were assessed and discussed through regular meetings with all research teams. Further, researchers have rich experience conducting qualitative research, which has been published.

Finally, it is essential to record the researcher’s personal notes and reflexes after each interview to examine the researcher’s biases, feelings, reflections, and values as a part of the data being collected (Lincoln & Guba, 1985; Munhall, 2001). The researcher maintained a journal to record the personal biases, assumptions, and reflections about the research throughout the study. Explication of personal beliefs enhances awareness of the potential judgments that may occur during data collection and analysis based on personal beliefs rather than on the actual data (Speziale & Carpenter, 2011).

### Data Analysis

The approach applied in analyzing the data was inductive qualitative content analysis (Elo et al., 2014). The analysis involved three main phases: preparation, organization, and reporting results. In the preparation phase, all interviews were immediately transcribed after being conducted. Then, the investigator read the transcript several times to understand the context, and then all-important statements were extracted. In the organization phase, all extracted statements were organized into open codes and then into manageable categories. One researcher performed coding and then assessed and discussed it by the remaining research team members to ensure accuracy and consistency.

In the third phase, the results were integrated into a theme and subthemes to describe the nurses’ experiences with COVID-19 “fully.” After that, researchers cross-checked their interpretations by experienced researchers to validate the trustworthiness of the findings. Finally, the participating nurses validated the performances (Colaizzi, 1978).

## Results

### Demographic Data

The sample included eight male and four female nurses with a mean age of 32.5 years (SD = 7.01; *R* = 26–52) and an average of 11.75 years of experience (SD = 7.07, *R* = 5–31). Eight of the nurses held a Bachelor of Science in Nursing (BSN), and 4 held a master’s degree (Table 2). Since the beginning of the outbreak in Jordan, the participating nurses have started caring for patients with COVID-19.

**Table 2.** Nurses’ Demographic Characteristics (*N* = 12).

Nurses (nurse no code)	Gender	Age (years)	Educational level	Years of experience
NH1	Male	35	BSN	14
NH2	Female	33	BSN	12
NH3	Female	34	Master’s	13
NH4	Male	32	BSN	12
NH5	Male	52	BSN	31
NH6	Male	35	Master’s	15
NK1	Male	26	Master’s	5
NK2	Female	32	BSN	12
NK3	Male	30	Master’s	10
NK4	Male	26	BSN	5
NK5	Female	26	BSN	5
NK6	Male	29	Master’s	7

*Note*. BSN = bachelor of science in nursing.

During data analysis, four major themes emerged which described the nurses’ experiences, abilities, and willingness regarding caring for patients with COVID-19: (1) uncertainty: negative and positive spheres; (2) social stigma; (3) front-line fighters; and (4) challenges (Table 3).

**Table 3.** Themes and Subthemes.

Theme	Subthemes
Uncertainty: negative and positive spheres	New experience
	Lack of training
Social stigma	
Front-line fighters	Empowering the main health caregiver
	Community acknowledgment
Challenges	Physical distress
	Psychological distress

#### Uncertainty: Negative and positive spheres

This theme reflects the nurses’ willingness to work with COVID-19 cases. On the one hand, some nurses had found uncertainty to motivate them to work with infected patients, as they considered this a social and religious commitment. Other nurses, however, had felt afraid because they were uncertain about the disease and its consequences. Two subthemes emerged from this theme: new experience and lack of training.

##### New experience

All of the nurses reported taking care of patients with COVID-19 as a new experience in terms of the disease process and its uncertainty.

One nurse said:

*As a nurse, I have dealt with H1N1influenza, the Middle East respiratory syndrome (MERS), and other pandemic diseases, but this virus has been spreading more rapidly than other viruses or epidemics we have dealt with, especially in Jordan. It was scary because it was new…“We used to deal with 40 or 50 cases per year with previous diseases, whereas we have dealt with 1200-1300 COVID-19 cases in 4-5 months. May God end this crisis." (N.11)*

Although the nurses expressed finding the disease terrifying, some reported that it had been a new experience they wanted to face.

One nurse said:

*I must say, the situation was terrifying for me at the beginning … Then, I told myself that I needed to go for it … I like to confront my fears … So, I told the head nurse that I wanted to volunteer. (N.12)*

Another nurse said:

*It’s scary to deal with something unknown… I didn’t mind taking the risk and facing the unknown, as this helps relieve the pressure and stress… After dealing with patients, the fear and stress have gone, thank God. (N.5)*

The fact that COVID-19 was a novel disease had led to unclear and continuously changing protocols, which had been challenging for the nurses:

*For us, there was no clear plan … It was only after we started receiving confirmed cases that we received policies from the Ministry of Health… (N.8)*

However, the nurses indicated that they had become more adaptive and relaxed with time and after knowing more about the disease.

One nurse said:

*In the beginning, it was terrifying, but with time, we slowly learned how to wear the uniform, follow precautionary measures, and deal with patients. (N.4)*

Another nurse indicated:

*Don’t forget that it was something new…. At first, we didn’t know how to deal with it, and we feared for ourselves and our families…. But now it has become a routine for us. (N.2)*

As a new experience with COVID-19, the nurses indicated that they had relied at first on following up on the updates on social media, which increased their uncertainty toward dealing with COVID-19 patients.

One nurse said:

*At the beginning of Corona, no one knew anything about the disease… and we looked for social media updates…. We were actually scared to work with the disease. (N. 5)*

Another nurse indicated

*We found difficulties in dealing with the disease because we heard a lot of unclear and misleading information from social media and the news. (N. 1)*

##### Lack of training

The majority of the nurses indicated that they had not received adequate training in caring for COVID-19-infected patients, which increased their uncertainty regarding the disease. The nurses reported relying on their readings to find out more about the disease.

One nurse said:

*Um… We do have the nursing department and Infection Control unit… They did hold lectures on precautionary measures… They taught us the basics … But we still felt curious… Although they explained some standardized procedures, this virus is new, and there might be additional procedures that we could learn. (N.7)*

Another nurse indicated:

*…. At first, we received suspected cases, and then our department started receiving confirmed cases… We [nurses] would read the WHO, and CDC (Centres for Disease Control and Prevention) reports, and we prepared ourselves. After we started receiving patients, the Infection Control Unit started holding training courses that would not last for more than 15 minutes about what we needed to wear and do to reduce contact with patients and so on. The rest we mostly knew from experience. (N.3)*

#### Social stigma

The participating nurses reported that people, including family members, had started to avoid them because they worked with COVID-19 cases. They shared several instances when they had felt socially isolated because others were avoiding them.

One nurse said:

*My neighbors were afraid of me. I had a neighbor whose kids always used to come over… but their mother isolated them from me completely after working with patients with COVID-19. I have not seen them for two months… It’s quite annoying. (N. 4)*

Some nurses also indicated that they had felt isolated from their colleagues and other healthcare workers even, which had negatively impacted them and their willingness to work with infected patients:

*We are the ones on the front-line. We do everything for the patients, and it’s still very challenging…. We are avoided by other hospital staff members even … As staff members dealing with infected patients, we are treated as outcasts… We have our external staircase for leaving the hospital…. We’re treated as outcasts even by doctors. (N.10)*

The participating nurses highlighted that social media had made the situation seem more complicated and terrifying and had increased the fears and stigma felt by society and other HCPs.

One nurse said:

*The media exaggerated the disease. We heard about the disease and were scared of it before reaching Jordan. But after dealing with patients, we realized that the disease and its complications were under control…. It’s just that it has become more of a phobia in society. It’s a phobia for us even though we are dealing with it. (N. 6)*

#### Front-line fighters

The participating nurses in this study indicated feeling that they were front-line fighters during the pandemic and that, as nurses, they played a significant role among other HCPs. Two subthemes emerged from this theme: empowering the main health caregiver and community acknowledgment.

##### Empowering the main health caregiver

The nurses indicated that they were the primary caregivers for COVID-19 patients most of the time. They reported that while they were the HCPs to spend most of their time with patients under normal circumstances, they were almost the only HCPs to remain with patients infected with COVID-19.

One nurse stated:

*Honestly, and I’m not just saying this because I’m a nurse myself, nurses dealt with COVID-19 cases from A-Z … We would even write prescriptions … The doctors would usually only do the swabs…We [nurses] provide them [patients] with the biggest service … from providing health education, reassurance, and care to be communicators between patients and doctors. (N.4)*

The nurses reported providing psychological support for patients as their significant role. They also indicated that patients with COVID-19 required extra psychological help because they were afraid of the disease.

One nurse said:

*We would be completely covered in protective clothing, so we couldn’t, for example, smile at the patient. I guess we could talk sweetly and provide psychological or emotional support … We would reduce patients’ panic towards this strange virus … I used to focus on providing patients with emotional and psychological support. With a few sweet words, I would make patients smile and calm them down, especially at the pandemic’s beginning. And thank God, things would go smoothly. (N. 5)*

The majority of the nurses felt that their experience in dealing with COVID-19-infected patients had positively influenced them and made them stronger and more confident nurses. They indicated that a few months of dealing with COVID-19 cases were equal to years of previous experience.

One nurse indicated:

*This experience has made us much stronger … It’s something we’ll tell our grandchildren about. It’s an experience I’m proud of… My mother always tells people about how her daughter is dealing with COVID-19-infected patients. She is proud rather than afraid. (N.9)*

Another nurse said:

*Honestly,… it’s a different experience… The three months we spent dealing with COVID-19 cases were equal to a year of experience…. We also feel more empowered …We have become more independent and work according to clear policies. (N.5)*

#### Community acknowledgment

One nurse said:

The nurses indicated that they felt more respected than before the pandemic and that their communities acknowledged the value of what they were doing.

*As nurses, we are more valued now … People’s view towards us changed because we were highly needed during the pandemic. Even the media … I mean, I was interviewed by an online newspaper … I felt interested in us nurses. I mean, in the beginning, they’d only host doctors, but then they started hosting nurses. (N.3)*

#### Challenges

This theme explains the significant challenges of working with COVID-19 cases, as reported by the nurses. These challenges can be categorized into physical and psychological challenges.

##### Physical challenges

The main physical challenges expressed by the nurses were related to wearing PPE. Having to work long shifts while wearing PPE was a frequently reported challenge. Physical challenges related to the isolation process itself were also reported.

One nurse mentioned:

*I mainly work the B/C shift. Seventeen continuous hours of working, wearing overalls, and hand-rubbing – look at how my hands are cracked from the sanitizers and the chemical materials … There were no breaks … and the biggest challenge was having to remain in the department for 17 hours. We were not allowed to leave. (N.9)*

Another nurse reported:

*The overalls we had to wear were the main challenge. Many of us were sensitive to the material, and it’s particularly annoying when you get hot …We’d get tired and sweaty …. It was also challenging when we’d have suspected cases and confirmed cases, and we’d have to wear a disposable gown over the overalls to go in to see patients … It was difficult to manage. (N.11)*

#### Psychological challenges

All of the interviewed nurses reported having experienced significant stress and anxiety when they had initially started working with COVID-19 cases, as the experience was new and the disease unknown.

One nurse stated:

*When they first told us that our department would be receiving COVID-19 cases, I was scared. There was a lot of anxiety and stress … When we started reading about the virus, and after the first suspected case was admitted, the fear and stress started gradually decreasing. But when the first confirmed case was admitted, our stress and anxiety levels increased, reflecting our behavior! We’d keep away from each other … wash our hands every 5 minutes, and constantly apply hand sanitizer … I mean, the first week was particularly stressful, but then we got used to the situation… Personally, I learned to live with it. (N.2)*

Further, the nurses expressed fear of transmitting the disease to their families as a significant psychological challenge. Many indicated that they had isolated themselves from their families, which, particularly for nurses with kids or sick parents, had caused a lot of stress.

One nurse said:

*The fear and stress were mainly related to avoiding contact with family… When they informed us that we’d be dealing with COVID-19 cases, I immediately told my mum that I’d be completely isolating myself … My biggest fear was transmitting the disease to my mother, as she is elderly and has diabetes and hypertension. She’s vulnerable, and that’s what scared me the most. (N.10)*

Another nurse said:

*My wife was pregnant … She was taking medications… and this reduced her immunity… I honestly isolated myself from her… I was afraid of going home and doing something wrong…. I still have these obsessive behaviors, especially when it’s someone close to you … I may sometimes forget to wipe my phone or wash my hands properly … I am just like other nurses … But I also have a family, and I could transmit the disease to them. I honestly isolated myself from my wife for a month and a half because she was pregnant and had poor immunity. I was honestly afraid for her. (N.7)*

## Discussion

The current study aimed to explore nurses’ experiences, abilities, and willingness to care for patients with COVID-19. The nurses’ descriptions of their lived experiences revealed their willingness and ability to care for patients with COVID-19. The nurses were aware of their responsibilities as front-line fighters to protect patients and society from the consequences of the disease. They reported facing several challenges before adapting to the situation and eventually learning to grow from it.

Initially, the nurses had experienced fear and uncertainty toward the new virus and its consequences, which is common among HCPs during biological disasters (Al Thobaity & Alshammari, 2020; Kackin et al., 2021; Khatatbeh, Alhalaiqa, et al., 2021; Liu et al., 2020; Shi et al., 2020; Xiang et al., 2020). The participating nurses consistently agreed that their experience with COVID-19 was new, and differed from other pandemic diseases they had dealt with previously. COVID-19; is unique in its high infectivity, transmissibility, fatality, and the absence of proven effective vaccines or therapies (Chew et al., 2020; Rathnayake et al., 2021). Therefore, nurses experienced fear and worry of being infected or spreading an infection to their families, friends, and society, consistent with previous Jordanian (Khatatbeh, Alhalaiqa, et al., 2021) and international studies (Ding et al., 2021; Halcomb et al., 2020; Kackin et al., 2021; Liu et al., 2020; Shi et al., 2020; Villar et al., 2021; Xiang et al., 2020). In turn, this had led some nurses to feel uncertain and reluctant, and others motivated to work with COVID-19-infected patients. However, despite the uncertainty and challenges they had faced, the nurses were willing to care for patients with COVID-19, given their sense of professional, religious, societal, and ethical/moral obligation to help patients in need. This strong sense of professional commitment among nurses (Fernandez et al., 2020; Ives et al., 2009; Khatatbeh, Alhalaiqa, et al., 2021; Liu et al., 2020; Rathnayake et al., 2021; Sun et al., 2020) and motivation to engage in new experience (Rathnayake et al., 2021) has been reported to outweigh their perceived barriers to their willingness and ability to work. Similar willingness to work amid the high risk of being infected was revealed in previous studies (Al-Dossary et al., 2020; Rathnayake et al., 2021; Villar et al., 2021). By contrast, unwillingness to work with COVID-19 was reported, with considerable proportions, by some studies conducted in Australia (44%) (Halcomb et al., 2020), the Philippines (17.2%) (Labrague & Santos, 2020), and China (22.8%) (Shi et al., 2020).

In addition, lack of knowledge and inadequate training about COVID-19 had increased the participating nurses’ uncertainty and reluctance toward working with COVID-19 patients. The nurses had felt the need to know more about protective planning since they were uncertain about the disease transmission modes, risks, prevention measures, and treatment. The participating nurse who had received training believed it had been brief and insufficient and that they had relied more on their readings, which can often be an unreliable source of information (Semaan et al., 2020). Similarly, lack of knowledge and inadequate training has been reported by HCPs in previous national (Khatatbeh, Alhalaiqa, et al., 2021) and international studies (Bhagavathula et al., 2020; Billings et al., 2021; Chegini et al., 2021; Ding et al., 2021; Rathnayake et al., 2021; Sun et al., 2020). Consistently, in Semaan et al. (2020) study, 92% of the HCPs personally searched for information about COVID-19, only 35% attended training on COVID-19, and only 19% perceived themselves to be thoroughly knowledgeable of providing care to women with COVID-19. In contrast, a higher percentage of Chines HCPs had adequate knowledge (Shi et al., 2020; Zhang et al., 2020) and correct practices regarding COVID-19 (Zhang et al., 2020). Also, a high percentage of nurses in Saudi Arabia reported excellent knowledge, preventive practices, and willingness toward COVID-19 (Al-Dossary et al., 2020). The more excellent knowledge was associated with more confidence in beating the virus, proper techniques (Zhang et al., 2020), and willingness to care for COVID-19 patients (Al-Dossary et al., 2020; Que et al., 2020; Shi et al., 2020). Thus, educational and training programs are essential for enhancing HCPs’ preparedness, willingness, and ability to effectively manage the COVID-19 outbreak (Al-Dossary et al., 2020; Ding et al., 2021; Liu et al., 2020; Que et al., 2020; Shi et al., 2020; Taylor et al., 2018; Zhang et al., 2020).

However, to fulfill their need for information about COVID-19 and reduce stress and uncertainty, the nurses in this study relied on reading the WHO and CDC reports. Though, they had also relied on following up the updates in social media and had emphasized its negative impact in increasing their uncertainty and fear toward COVID-19, consistent with other studies (Billings et al., 2021; Galehdar et al., 2021; Iheduru-Anderson, 2021; Khatatbeh, Alhalaiqa, et al., 2021; Semaan et al., 2020). Interestingly, some HCPs were paying less attention to media and news about COVID-19 to avoid its negative impacts, which was helpful to be calm, rational, and focusing on their work (Ding et al., 2021; Kackin et al., 2021). HCPs commonly use personal searches for information through informal networks or social media (Billings et al., 2021; Khatatbeh, Alhalaiqa, et al., 2021; Semaan et al., 2020), although the accessed information may often be unreliable (H. Li et al., 2020; Semaan et al., 2020). Therefore, professional channels for sharing information about the disease should be established to ensure nurses’ timely access to accurate and updated information (Semaan et al., 2020; Xiang et al., 2020).

Further, nurses in this study indicated that the unclear and constantly changing COVID-19 protocols and guidelines in the early stage had increased their feelings of uncertainty and confusion, creating a challenge for them, supporting previous studies (Billings et al., 2021; Chegini et al., 2021; Ding et al., 2021; Rathnayake et al., 2021). However, with time, as the participating nurses gained more knowledge and had more apparent protocols and guidelines to follow, they began to feel familiar with the disease. They became more adaptive, capable, confident, and relaxed. Others reported a similar experience (Chau et al., 2021; Ding et al., 2021). Thus, it is essential to educate and train nurses early about the emergency strategies, protective measures, what is expected from them, and standardized procedures to enhance their ability to respond effectively to the outbreak (Al-Dossary et al., 2020; Ding et al., 2021; Liu et al., 2020; Rathnayake et al., 2021; Shi et al., 2020; Taylor et al., 2018; Zhang et al., 2020).

As front-line HCPs during the pandemic, the nurses in this study had initially experienced stress and anxiety due to the novel and life-threatening nature of the disease. Previous studies have reported similar psychological impacts (Ding et al., 2021; Halcomb et al., 2020; Kackin et al., 2021; Karimi et al., 2020; Khatatbeh, Alhalaiqa, et al., 2021; Labrague & Santos, 2020; Lai et al., 2020; Liu et al., 2020; Temsah et al., 2020; Villar et al., 2021; Xiang et al., 2020). The HCPs’ exposure to life-threatening situations correlates positively with their levels of psychological stress (Braquehais et al., 2020; Hawari et al., 2021). One of the main factors that increased participating nurses’ stress and fear of transmitting the disease was living with vulnerable family members such as children, pregnant women, or sick and old parents, consistent with other national and international studies (Braquehais et al., 2020; Ding et al., 2021; Hawari et al., 2021; Khatatbeh, Alhalaiqa, et al., 2021; Liu et al., 2020; Rathnayake et al., 2021; Shi et al., 2020). This factor was a commonly reported psychological challenge impacting the participating nurses’ willingness and ability to work during the pandemic. However, consistent with many studies (Braquehais et al., 2020; Ding et al., 2021; Liu et al., 2020; Shi et al., 2020), the nurses had volunteered to work with COVID-19 cases despite their stress and anxiety.

Many nurses had quarantined themselves from their families and others to prevent the risk of transmission, which had exacerbated their feelings of anxiety, stress, and social isolation. Social isolation is a problematic issue, particularly with the social gathering habits of family members in Jordan. Consistently, social isolation was reported as one of the most significant psychological challenges in another Jordanian study (Khatatbeh, Alhalaiqa, et al., 2021). HCPs have reported similar psychological impacts of quarantined in many studies (Braquehais et al., 2020; Brooks et al., 2020; Chau et al., 2021; Ding et al., 2021; Huang et al., 2020; Iheduru-Anderson, 2021; Kackin et al., 2021).

In addition, the participating nurses reported that as front-line caregivers, they and their families had felt stigmatized and avoided by society and other HCPs, which is also in line with the Jordanian (Khatatbeh, Alhalaiqa, et al., 2021) and other studies (Brooks et al., 2020; Chew et al., 2020; Ding et al., 2021; Kackin et al., 2021; Y. Kim, 2018; W. Li et al., 2021; Rathnayake et al., 2021). The nurses in this study highlighted the role of social media’s exaggeration of COVID-19 in raising fears and social stigma against nurses who were caring for infected patients. Exaggerated and misleading information about COVID-19 on social media may create fear (González-Padilla & Tortolero-Blanco, 2020) and propagate stigma (Billings et al., 2021; Cho et al., 2020; Mostafa et al., 2020). The perceived social stigma may escalate HCPs’ psychological stress and burnout (Brooks et al., 2018; Chew et al., 2020; Ding et al., 2021; Kackin et al., 2021; Khatatbeh, Alhalaiqa, et al., 2021; W. Li et al., 2021; Ramaci et al., 2020), hence impacting the willingness and ability to care for patients (Brooks et al., 2018; Martin, 2011; Que et al., 2020; Ramaci et al., 2020). The social stigma with COVID-19 is a significant predictor of compassion fatigue, compassion satisfaction, and burnout among HCPs (Ramaci et al., 2020). Therefore, the implementation of stress management programs, such as copying and resilience programs, is necessary for alleviating the high levels of psychological stress among HCPs. Especially for nurses working directly with COVID-19-infected patients, these programs will improve HCPs’ psychological skills and coping abilities and will better enable them to fight the pandemic (Preti et al., 2020; Ramaci et al., 2020).

The availability of PPEs was significant for providing direct care for COVID-19 patients and reducing the associated stress. Though, nurses in this study were physically challenged, particularly with the heavy workloads and long shifts. Having to wear full PPE during long shifts in the isolation wards had caused the nurses physical discomfort (sweating, skin remarks, and cracks) and fatigue and had impacted their ability to work. Wearing PPE had made carrying out tasks such as providing patients with routine direct care, vigilant monitoring, communication, and psychological support challenging and stressful, which is consistent with other studies (Ding et al., 2021; Khatatbeh, Alhalaiqa, et al., 2021; Liu et al., 2020; Rathnayake et al., 2021; Sun et al., 2020; Villar et al., 2021). Meanwhile, other studies have reported that providing nurses with adequate PPE supplies and a safe work environment reduces their fear and uncertainty (Ding et al., 2021; Khatatbeh, Alhalaiqa, et al., 2021; Liu et al., 2020) and increases their willingness and ability to work during infectious disease outbreaks (Aoyagi et al., 2015; Ding et al., 2021; Halcomb et al., 2020; Martin, 2011; Taylor et al., 2018). Significant personal physical safety concerns caused many nurses to consider resigning from their work (Halcomb et al., 2020).

The participating nurses reported that sharing their experiences and feelings helped them adapt to the situation and encouraged them to fight the disease. Further, family members, friends, and colleagues had also gradually overcome their fear of infection and become a source of support for the nurses. Similar sources and impact of social support were reported by Jordanian and other nurses involved in similar outbreaks (Chen et al., 2020; Ding et al., 2021; Khatatbeh, Alhalaiqa, et al., 2021; Y. Kim, 2018; Labrague & Santos, 2020; Liu et al., 2020; Sun et al., 2020; Villar et al., 2021). Further, nurses in this study reported adopting positive coping strategies, including positive appraisal, problem-solving, planning activities, seeking information, seeking social support, praying, and changing environments. These positive strategies included appraising and accepting the existence of the disease, focusing on the possible ways to control the problem and its related stressors, reading, and sharing updated information and guidelines, adopting infection control measures in treating patients, self-isolating or quarantining, and protecting themselves and others from the risk of infection. Such strategies are effective in stressful situations (Chew et al., 2020; Ding et al., 2021; Kackin et al., 2021; Khalid et al., 2016; Labrague & Santos, 2020; X. Li et al., 2021; Si et al., 2020; Sun et al., 2020; Villar et al., 2021; Yu et al., 2020). In contrast, a previous Jordanian study reported maladaptive behaviors by nurses such as heavy smoking and coffee drinking (Khatatbeh, Alhalaiqa, et al., 2021). Meanwhile, previous studies have reported other coping mechanisms which can relieve stress and enhance mental health among nurses, such as avoiding negative media, carrying out breathing exercises, following religious activities, focusing on distractions, and using music meditation (Ding et al., 2021; Kackin et al., 2021; Rathnayake et al., 2021; Sun et al., 2020; Villar et al., 2021). Positive coping strategies and sufficient social support are essential for alleviating psychological stress and mental problems (Chew et al., 2020; Ding et al., 2021; Labrague & Santos, 2020; Rathnayake et al., 2021; Si et al., 2020; Villar et al., 2021; Yu et al., 2020). Unfortunately, similar to nurses in other studies (Ding et al., 2021; Iheduru-Anderson, 2021; Kackin et al., 2021; Liu et al., 2020; Rathnayake et al., 2021), the nurses in this study had not received professional psychological counseling or adequate support from managers. This implies the need to strengthen support systems and provide psychological counseling to allow nurses to adapt effectively during disease outbreaks (Iheduru-Anderson, 2021; Kackin et al., 2021; Liu et al., 2020; Rathnayake et al., 2021; Sun et al., 2020).

In line with the findings of Liu et al. (2020), the nurses in the present study considered themselves front-line fighters during the COVID-19 pandemic, as they were the only HCPs to work long shifts in the isolation wards to provide patients with nursing care, psychological support, and help in carrying out daily living activities and communicating with families. Consistent with other nurses’ reported experiences during infectious disease outbreaks (Ding et al., 2021; Galehdar et al., 2021; Kackin et al., 2021; H. S. Kang et al., 2018; Rathnayake et al., 2021; Sun et al., 2020), the participating nurses’ skills and abilities of communication, management of emergencies, and care were enhanced with time. The nurses in our study felt their strenuous efforts had been acknowledged and appreciated by their patients, families, friends, and communities, making them feel more respected than before the pandemic.

Overall, the experience of working with COVID-19 cases had allowed the nurses to grow and become more robust nurses, which has been observed among nurses working during other infectious disease outbreaks (Ding et al., 2021; Galehdar et al., 2021; Kackin et al., 2021; H. S. Kang et al., 2018; Y. Kim, 2018; Rathnayake et al., 2021; Sun et al., 2020). Being primary caregivers to COVID-19 patients had allowed the nurses to develop their role-specific knowledge and skills, thus making them feel more willing, powerful, and confident to work in similar situations in the future. Further, the nurses reported that fulfilling their professional responsibility, providing high-quality care for patients with COVID-19, and receiving acknowledgment from others had given them a sense of achievement and pride, supporting previous findings of outbreaks (Ding et al., 2021; Galehdar et al., 2021; Kackin et al., 2021; H. S. Kang et al., 2018; Khatatbeh, Alhalaiqa, et al., 2021; Y. Kim, 2018; Rathnayake et al., 2021; Sun et al., 2020; Villar et al., 2021). Similarly, in the study of Sun et al. (2020), working with COVID-19 cases allowed the participating nurses to grow professionally and psychologically.

Self-empowerment, psychological growth, and confidence are significant outcomes of the experience of working with COVID-19 and can positively impact nurses’ willingness and ability to deal with future outbreaks. However, to optimize nursing workforce retention, sustainability, and care quality, nurses who are caring for COVID-19 patients must receive early training and education about the risks, and protective and personal safety measures. Early training, having confidence in one’s role-specific skills, and being knowledgeable about risks, protective measures, and personal safety are significant predictors of nurses’ willingness to work during disease outbreaks (Aoyagi et al., 2015; Shi et al., 2020). In addition, they must receive timely psychosocial assessment, and support from their health organization. Clear protocols and regulations about COVID-19 are very important to fight the virus. Our study was conducted during the early phase of the COVID-19 outbreak when the new protocols were changing frequently and rapidly.

## Limitations

Due to the implemented outbreak safety measures, the authors could not conduct face-to-face and focus group interviews. However, video interviews were conducted online using the Zoom software application to obtain adequate verbal and nonverbal data. The use of focus group interviews would have facilitated interaction and enriched the findings. Also, using a qualitative design among a small sample from two hospitals limits the accuracy and generalizability of the results.

## Implications for Nursing Practice and Future Studies

Our study findings provide baseline data for authorities and administrators to plan effective interventions to improve the national nursing workforce’s emergency readiness and response to COVID-19 and future pandemics. There is a need to plan and implement effective nursing education and training programs on emergency strategies, protective measures, expected roles in pandemic responses, and standardized procedures to enhance nurses’ abilities to respond effectively to an outbreak. Establishment and efficient dissemination of clear protocols and guidelines on COVID-19 at early stages can decrease nurses’ feelings of uncertainty and confusion.

The nurses demonstrated their responsibility and commitment as front-line fighters against COVID-19, yet they faced several physical and psychological challenges. The study findings encourage healthcare institutions to implement psychological counseling and support interventions for the frontline nurses at an early phase in future outbreaks to alleviate and manage their psychological stress. Also, the experienced social stigma-related stress among nurses in this study implies the need to reinforce support systems and networks, collaborate with community organizations to address stigma, and increase community and HCPs’ awareness and skills to fight stigma. Further, considering the role of exaggerated and misleading information about COVID-19 on social media in raising fears and social stigma against nurses caring for infected patients, it is essential to establish professional channels for information-sharing to ensure timely access to accurate and updated information and guidelines on COVID-19.

This study represents nurses’ experiences of caring for patients with COVID-19, representing a subset of HCPs. Studies that examine nurses’ willingness and ability to work with COVID-19 patients, including those not previously involved in COVID-19 care, are recommended. Future studies that explore the experiences of administrative nurses and other HCPs are also recommended. Moreover, future studies are required for a greater understanding of COVID-19-related social stigma among society and HCPs.

## Conclusion

This study identified various vital themes related to nurses’ experiences, willingness, and ability to care for patients with COVID-19. Caring for COVID-19 patients has been a positive experience for nurses in Jordan. Initially, the participating nurses experienced many challenges which impacted their willingness and ability to work with COVID-19 cases, including lack of certainty, psychological and physical stress, and social stigmatization. Despite this, the nurses remained committed to helping those in need, which gradually allowed them to grow professionally and psychologically and become more knowledgeable, skillful, powerful, and confident nurses. Consequently, this increased their willingness to work under similar circumstances in the future. Providing early training, psychological assessment and counseling, adequate social support, and promoting practical coping skills are essential steps for ensuring the mental well-being of nurses and increasing their willingness and ability to fight COVID-19.
